# High In-Hospital Mortality Incidence Rate and Its Predictors in Patients with Intracranial Hemorrhage Undergoing Endotracheal Intubation

**DOI:** 10.3390/neurolint13040064

**Published:** 2021-12-01

**Authors:** Hong-Khoi Vo, Cong-Hoang Nguyen, Hoang-Long Vo

**Affiliations:** 1 Neurology Center, Bach Mai Hospital, Hanoi 100000, Vietnam; hoangnc92@gmail.com; 2 Department of Neurology, Hanoi Medical University, Hanoi 100000, Vietnam; 3 Department of Neurology, VNU Medical School, Vietnam National University, Hanoi 100000, Vietnam; 4 Institute for Preventive Medicine and Public Health, Hanoi Medical University, Hanoi 100000, Vietnam; vohoanglonghmu@gmail.com

**Keywords:** endotracheal intubation, in-hospital mortality, intracranial hemorrhage

## Abstract

(1) Background: The goal of this study was to determine the incidence of in-hospital mortality and to investigate its predictors in patients with a primary intracranial hemorrhage (ICH) undergoing endotracheal intubation. (2) Methods: This retrospective study, between July 2018 to July 2019, recruited patients who were diagnosed with a primary ICH and who were intubated during treatment in our institution. The outcome variable was in-hospital mortality, known as 30-day mortality, in patients with ICH undergoing endotracheal intubation. Multivariable analyses were performed to identify the prediction of in-hospital mortality. (3) Results: A total of 180 patients with ICH undergoing endotracheal intubation were included, with a mean (SD) age of 62.64 (13.82) years. A total of 73.33% were female, and 71.11% of the patients were indicated for intubation due to neurological reasons. The in-hospital mortality rate, following endotracheal intubation, was 58.33%. In a reduced model using a stepwise backward selection strategy with *p* values < 0.2, independent predictors of in-hospital mortality were brain herniations on cranial CT scans (OR: 10.268, 95% CI: 2.749–38.344), lower Glasgow coma scale (CGS) scores before intubation (OR: 0.614, 95% CI: 0.482–0.782), and the loss of the vertical oculocephalic reflex before intubation (OR: 6.288, 95% CI: 2.473–15.985). Conclusions: The in-hospital mortality rate was comparable to that in the early evidence, but was significantly higher compared to recent reports. We infer that brain herniations on cranial CT imaging, lower CGS scores before intubation, and the loss of the vertical oculocephalic reflex before intubation could be used to approximately predict in-hospital mortality in patients with primary ICH undergoing endotracheal intubation. These considerations can help guide clinical decisions and community stroke discussions.

## 1. Introduction

Intracranial hemorrhage (ICH) is a significant medical event that accounts for up to 15% of strokes [1]. The incidence of ICH is approximately 25 per 100,000 person-years, and it has a mortality of 40% within one month of presentation [1]. The incidence of ICH is substantially variable, and may be lower across countries and ethnicities, such as 8–15% in western countries, such as the USA, the UK, and Australia [2,3]. Moreover, a 47% increase in the absolute number of hemorrhagic strokes was estimated globally between 1990 and 2010, as stated by the Global Burden of Disease 2010 study [4]. Notably, a systematic review from 56 population-based studies suggested that, compared to high-income countries, low- and middle-income countries had a twice-higher incidence of primary ICH (22 vs. 10 per 100,000 person-years) from 2000–2008 [5].

According to the most recent data in Vietnam—a lower- and middle-income country in Southeast Asia—the overall prevalence of strokes between 2013 and 2014 was 1.62%, with the highest incidence in Can Tho (4.81%) and the lowest in Gia Lai (0.36%). A study across Vietnam by Le Van Thinh (2008) in 78 provincial, city, and central hospitals suggested that the mortality rates from strokes in health facilities were 4.4% in the north, 4.1% in the central, and 5% in the south regions of Vietnam [6]. It was estimated that 200,000 Vietnamese suffered from strokes every year; up to half of these died, and 90% of stroke survivors lived with neurological and motor sequelae [7]. Nevertheless, national data on ICH has been not available in this country, which still requires up-to-date evidence from different major health centers.

Clinical and laboratory factors for predicting mortality are extremely important and can influence the treatment attitude of doctors. While several poor prognostic factors in ICH patients, reported in previous studies, were increasing age, decreasing Glasgow coma scale (GCS) scores, increasing ICH volumes, the presence of intraventricular hemorrhages, a deep/infratentorial ICH location, and prior antithrombotic (antiplatelet and anticoagulant) drugs [8,9,10], potential predictors for in-hospital mortality in patients with ICH indicated for endotracheal intubation, from the perspective of the Vietnam hospital-based evidence, remain unknown. To the best of our knowledge, the studies in the ICH patients who were intubated are scarce, and most of them are from a decade ago. Especially in Vietnam, a developing country with many barriers for patients in the hospital care system and where the proportion of ICH incident cases is high and tends to increase over time, no reports have been published. The lack of studies focusing on ascertaining poor risk factors specifically pertaining to death after endotracheal intubation among ICH patients poses further comprehensive investigation. To fill this knowledge gap, this study sought to determine the incidence of in-hospital mortality and to investigate its predictors in patients with primary intracranial hemorrhage undergoing endotracheal intubation. Thereby, our results might have some implications for establishing appropriate treatment strategies for decreasing mortality and disability rates and improving clinical outcomes.

## 2. Methods

### 2.1. Patient Population

From July 2018 to July 2019, a cohort of all consecutive patients admitted with the diagnosis of a primary ICH to a large hospital and intubated during treatment in an academic medical center (Neurology Center, Bach Mai Hospital, Hanoi, Vietnam) was investigated.

The exclusion criteria were secondary ICH caused by trauma, tumors, arteriovenous malformations or aneurysms, terminal illnesses or end-stage diseases such as severe heart failure, end-stage renal diseases, or decompensated cirrhosis.

### 2.2. Variable Definitions

All potential predictive factors have been documented in this study on the basis of clinical assessments and literature analyses.

#### 2.2.1. Outcome Variable

The outcome variable was in-hospital mortality, known as 30-day mortality, in patients with intracranial hemorrhage undergoing endotracheal intubation.

#### 2.2.2. Collected Potential Variables

The collected potential variables include: patient-related personal data (age, gender, medical history and comorbidities, and BMI), admission clinical data (time intervals between onset and patients’ arrival to the health facility, Glasgow coma scale (GCS) scores, extent of motor paralysis, facial nerve paralysis, Babinski signs, pupillary reactivity, pupillary light reactivity, dysphagia, headaches, stiff necks, and seizures), admission laboratory testing (hypernatremia/hyponatremia, increased white blood cell counts, hypercoagulable disorders, and admission hyperglycemia), computed tomography (CT) imaging (intracerebral hematoma location, volume of the intracerebral hematoma, compression of the ventricular system, midline shifts, brain herniations, hemorrhagic ventricular dilatation, pneumonia, and recurrence of bleeding), and clinical data before intubation (time intervals from clinical onset to intubation, blood pressure > 180/100 mm Hg, GCS scores, vertical and horizontal oculocephalic reflexes, pupillary reactivity, pupillary light reflexes, and indication for endotracheal intubation).

### 2.3. Data Analysis

All data was first subjected to a visual inspection for coding errors, outliers, or funky distributions. The in-hospital mortality patient group and the in-hospital survival patient group were compared according to admission clinical data, admission laboratory testing, CT imaging features, and clinical data before intubation. Categorical variables were described using proportions, while continuous variables were expressed as summary statistics. The *χ*^2^ test and the Fisher exact test were used to compare proportions, whereas the t test or the Mann–Whitney test was used, as appropriate, to compare continuous variables. Next, we applied the multivariable logistic regression technique to model the probability of in-hospital mortality with a range of potential variables (full model). Then, to obtain the final reduced model, including predictors of in-hospital mortality in ICH patients undergoing endotracheal intubation, we applied a stepwise backward selection strategy with *p*-values < 0.2 for the previous full model. A *p*-value < 0.05 was considered to be statistically significant. All statistical analyses were performed with a software program (Stata^®^ 15; StataCorp LLC, College Station, TX, USA).

## 3. Results

In total, 180 patients with intracranial hemorrhage undergoing endotracheal intubation were included in the study, with a mean (SD) age of 62.64 (13.82) years. The in-hospital mortality in patients with intracranial hemorrhage who were intubated was 58.33%.

Among the 180 study patients, the mean time interval between onset and the patients’ arrival to our center was 0.88 (±1.68) hour, and BMI was 20.78 (±2.97) (kg/m^2^). A total of 12.22% of the patients had GCS scores of 8 or less (Table 1). A comparison of hospital admission data between in-hospital mortality patients and in-hospital survival patients is presented in Table 1. In-hospital mortality patients and in-hospital survival patients were similar in age, BMI, medical history/comorbidity, time intervals between onset and arrival to the health facility, GCS scores, extent of motor paralysis, facial nerve paralysis, Babinski signs, pupillary reactivity, pupillary light reactivity, dysphagia, headaches, and seizures. However, the proportion of in-hospital mortality was significantly higher in males than it was in females (*p* = 0.040). Stiff necks were more common in in-hospital mortality patients (*p* = 0.024).

Among them, 16.67% had hypernatremia/hyponatremia, 66.67% had increased white blood cell counts, 15.00% had hypercoagulable disorder, and 10.56% had hyperglycemia at admission. Table 2 shows no significant difference in admission laboratory testing amongst the in-hospital survival and in-hospital mortality patients with ICH.

Computed tomography (CT) imaging of patients with intracranial hemorrhage undergoing endotracheal intubation is shown in Table 3. More than half of the patients in the mortality group had large hematoma volumes (58.10%), while this figure was 25.33% in survivors (*p* < 0.001). There were significant differences in the CT imaging of the compression of the ventricular system (*p* = 0.020) and brain herniation (*p* < 0.001), which was documented commonly in mortality patients. Mortality patients had the higher proportion of recurrence of bleeding compared to survivors (31.43% vs. 16.00%) (*p* = 0.018). However, the proportion of those having chest CT imaging of pneumonia was significantly higher among in-hospital survival patients (*p* = 0.015).

Most patients were indicated for intubation due to neurological reasons (71.11%), which was significantly higher in in-hospital mortality patients (87.62%) compared to survival patients (48.00%). Table 4 provides the main clinical data before intubation. Clinical data before intubation, including blood pressure ≥180/100 mmHg, GCS scores, vertical and horizontal oculocephalic reflexes, pupillary reactivity, and pupillary light reflex, were significantly different between in-hospital mortality patients and in-hospital survival patients. The mean GCS score and the time interval from clinical onset to intubation was 8.31 (±2.88) and 3.68 (±4.08) hours, respectively. Survivors had significantly higher GCS scores (*p* < 0.001) and more prolonged mean time intervals from clinical onset to intubation (*p* = 0.032).

In the full model with multivariate logistic regression (Table 5), admission hyponatremia (OR: 10.364, 95% CI: 1.565–68.615), brain herniations on cranial CT scans (OR: 19.807, 95% CI: 3.255–120.522), lower CGS scores before intubation (OR: 0.518, 95% CI: 0.361–0.744), and negative vertical oculocephalic reflexes before intubation (OR: 6.336, 95% CI: 1.848–21.720) were found to be significantly associated with in-hospital mortality after ICH.

As shown in Table 6, the results analyzed using a stepwise backward selection strategy with *p* values < 0.2 were similar to the results in Table 5. Independent predictors of in-hospital mortality, which were obtained from the final reduced model, were brain herniations on cranial CT scans (OR: 10.268, 95% CI: 2.749–38.344), lower CGS scores before intubation (OR: 0.614, 95% CI: 0.482–0.782), and negative vertical oculocephalic reflexes before intubation (OR: 6.288, 95% CI: 2.473–15.985).

## 4. Discussion

In this study, which included 180 patients with intracranial hemorrhages undergoing endotracheal intubation, the incidence of in-hospital mortality was 58.33%. Our incidence rate was comparable to that reported in previous hospital-based evidence [11,12,13,14,15]; however, such similar rates were in studies reported prior to 2000. A study by Lioutas V.-A (2018) of a recent cohort of all consecutive patients admitted with acute spontaneous ICH from 2000 to 2014 documented that 38.5% of intubated patients died during hospitalization [16]. Our incidence rate was significantly higher than Lioutas V.-A’s reported rate. Here, a suitable explanation might be the difference in exclusion criteria between the two studies, specifically that Lioutas V.-A excluded admissions that were less than 48 h in duration. In fact, some evidence also revealed that half of all deaths occurred in the first 48 h [17,18,19]. To our knowledge, although medical progress has been made in years past, individuals with ICH requiring intubation have very poor outcome prognoses, without significant improvement over time.

In agreement with the available literature, we found endotracheal intubations were indicated mostly for neurological reasons in 71.11% of all patients. Previous authors found that the rationale for intubation had no predictive value for mortality in ICH patients [20]. According to our present data, among the mortality group, 87.62% were indicated for intubation due to neurological reasons, while this figure was 12.38% for respiratory reasons. Herein, a range of risk factors was associated with higher rates of in-hospital mortality in ICH patients in our analysis. Conforming to our final reduced model with logistic regression, three factors, including brain herniation on cranial CT imaging, lower CGS scores before intubation, and negative vertical oculocephalic reflexes before intubation, were significantly associated with in-hospital mortality in primary ICH patients, following endotracheal intubation.

Hyponatremia, a common electrolyte abnormality in spontaneous ICH patients, seems to be a preexisting condition, rather than on that develops acutely, and is especially independently associated with admission anemia [21]. Hyponatremia has gained wide recognition in Joji B Kuramatsu’s study (2014) as an independent predictor of in-hospital mortality with a fairly high prevalence in spontaneous ICH patients [21]. Not unexpectedly, our present analysis was also in line with existing data. In full models with multivariate logistic regression, the odds of in-hospital mortality were 10.364 times higher in hyponatremia patients, compared to those having normal serum sodium at admission.

GCS score, a component of numerous trauma scoring systems, is most commonly used to describe the level of consciousness in a person following a traumatic brain injury. We found that a low GCS score, evaluated at the time before intubation, was an independent predictor for in-hospital mortality in patients with ICH. For every GCS score increase of one, the odds of in-hospital death decreased by 0.614%. A study by Rutten-Jacobs also reported that a lower average GCS score was associated with an increased risk of early mortality in patients with ICH [22]. An earlier occurrence of consciousness disturbance and more severe conditions may, therefore, suggest greater damage to the involved cerebral structures, as well as a greater mortality rate.

Brainstem dysfunction was previously mentioned by the authors as a significant prognostic sign of mortality in ICH patients undergoing endotracheal intubation [11,12,13,14]. The final reduced model in the current study indicated that the negative vertical oculocephalic reflex before intubation increased the odds of in-hospital death by 6.288-fold. Previous reports have shown consistencies in the results for the vertical oculocephalic reflex. Other brainstem signs have also been found to be prognostic contributors for mortality, such as the loss of the horizontal oculocephalic reflex [11,13] and the loss of the bilateral pupillary light reflex [14]; however, these two variables were not statistically significant in this study.

Cranial CT is an excellent and convenient tool for the diagnosis and prognosis of cerebral hemorrhage. All of our patients received a cranial CT scan at the beginning of admission in case the patient was clinically severe. Brain herniations on cranial CT scans were indicated in existing reports to be related to an increase in the likelihood of in-hospital mortality [23,24]. Our result was in line with previous evidence. The presence of a brain herniation on the cranial CT scan resulted in a 10.268-fold increase in the likelihood of in-hospital mortality and an incidence rate of in-hospital mortality, in those with a brain herniation, of 89.80%. Because of the popularity of cranial CT scans in Vietnam’s medical institutions, the present findings are valuable for the physicians in the treatment and prognosis of poor outcomes in post-intubation ICH patients.

Though in-hospital mortality remains an important outcome for ICH patients following endotracheal intubation and is easily examined by neuroradiologists, this is, to the best of our knowledge, the most comprehensive report available in developing countries and in Southeast Asia that analyzes prognostic factors for in-hospital mortality in post-intubation ICH patients. The strengths of our study includes its relatively large sample size with a broad variety of clinical, laboratory, and CT imaging potential risk factors. However, there were three main limitations in our study. First, all study patients were obtained from a single institution. Second, our results might have been influenced, to some extent, by the lack of clinical data before the patient was referred to our hospital, as some patients were referred from another hospital to our center. Third, a detailed subgroup analysis was not performed for hemorrhages at different locations, nor for hematoma volumes, in this study.

## 5. Conclusions

The in-hospital mortality rate was comparable to that of the early evidence, but was significantly higher compared to recent reports. We infer that brain herniations on cranial CT imaging, lower CGS scores before intubation, and the loss of the vertical oculocephalic reflex before intubation could be used to approximately predict in-hospital mortality in patients with primary ICH undergoing endotracheal intubation.

## Figures and Tables

**Table 1:** Clinical data at admission for the in-hospital survival and in-hospital mortality patients with ICH.

Variables	All Patients (*n* = 180)	In-Hospital Mortality		*p*-Value
		No (*n* = 75)	Yes (*n* = 105)	
	Count (% of Total)	Count (% of Total)	Count (% of Total)	
Gender				0.040 ^C,^*
Male	132 (73.33)	61 (81.33)	71 (67.62)	
Female	48 (26.67)	14 (18.67)	34 (32.38)	
Hypertension	127 (70.56)	54 (72.00)	73 (69.52)	0.719 ^C^
History of alcohol abuse	38 (21.11)	15 (20.00)	23 (21.90)	0.758 ^C^
Diabetes	18 (10.00)	7 (9.33)	11 (10.48)	0.801 ^C^
History of cerebrovascular accident	29 (16.11)	15 (20.00)	14 (13.33)	0.230 ^C^
GCS score				0.323 ^C^
≤8	22 (12.22)	8 (10.67)	14 (13.33)	
9–12	81 (45.00)	30 (40.00)	51 (48.57)	
13–15	77 (42.78)	37 (49.33)	40 (38.10)	
Extent of motor paralysis				0.476 ^F^
Hemiplegia	159 (88.33)	67 (89.33)	92 (87.62)	
Quadriplegia	16 (8.89)	5 (6.67)	11 (10.48)	
No paralysis	5 (2.78)	3 (4.00)	2 (1.90)	
Facial nerve paralysis				1.000 ^F^
Central facial palsy	154 (85.56)	64 (85.33)	90 (85.71)	
Peripheral facial nerve palsy	16 (8.89)	7 (9.33)	9 (8.57)	
No paralysis	10 (5.56)	4 (5.33)	6 (5.71)	
Babinski sign				0.513 ^F^
Unilateral positive	150 (83.33)	65 (86.67)	85 (80.95)	
Bilateral positive	25 (13.89)	9 (12.00)	16 (15.24)	
Negative	5 (2.78)	1 (1.33)	4 (3.81)	
Pupillary reactivity				0.111 ^F^
Unilateral mydriasis	12 (6.67)	6 (8.00)	6 (5.71)	
Bilateral mydriasis	6 (3.33)	0 (0.00)	6 (5.71)	
No mydriasis	161 (89.44)	69 (92.00)	92 (87.62)	
Pupil contraction	1 (0.56)	0 (0.00)	1 (0.95)	
Pupillary light reflex				0.297 ^F^
Positive	162 (90.00)	69 (92.00)	93 (88.57)	
Negative	17 (9.44)	5 (6.67)	12 (11.43)	
N/A	1 (0.56)	1 (1.33)	0 (0.00)	
Dysphagia				0.696 ^C^
No	41 (22.78)	16 (21.33)	25 (23.81)	
Yes	139 (77.22)	59 (78.67)	80 (76.19)	
Headache				0.296 ^F^
No	63 (35.00)	22 (29.33)	41 (39.05)	
Yes	117 (65.00)	53 (70.67)	64 (60.95)	
Stiff Neck				0.024 ^C,^*
No	51 (28.33)	28 (37.33)	23 (21.90)	
Yes	129 (71.67)	47 (62.67)	82 (78.10)	
Seizure				0.215 ^C^
No	164 (91.11)	66 (88.00)	98 (93.33)	
Yes	16 (8.89)	9 (12.00)	7 (6.67)	
	**Mean** ± **SD (IQR)**	**Mean** ± **SD (IQR)**	**Mean** ± **SD (IQR)**	
Time interval between the onset and arrival to our center (hours)	0.88 ± 1.68 (0.04–12)	1.00 ± 2.01 (0.04–12)	0.80 ± 1.40 (0.04–8)	0.9802 ^M^
Patient age (years)	62.64 ± 13.82 (24–93)	63.56 ± 11.95 (24–89)	61.99 ± 13.76 (30–93)	0.2271 ^T^
BMI (kg/m^2^)	20.78 ± 2.97 (14.6–34.6)	21.00 ± 3.04 (16–34.6)	20.62 ± 2.99 (14.6–28)	0.5650 ^M^
GCS score	11.68 ± 2.71 (3–15)	12.05 ± 2.58 (3–15)	11.41 ± 2.78 (3–15)	0.0907 ^M^

SD: standard deviation; IQR: Interquartile range; ^F^: Fisher’s exact test; ^C^: *χ*^2^ test; ^T^: *t*-test; ^M^: Mann–Whitney U test, *: significant at 0.05.

**Table 2:** Admission laboratory testing in the in-hospital survival and in-hospital mortality patients with ICH.

Variables	All Patients (*n* = 180)	In-Hospital Mortality		*p*-Value
		No (*n* = 75)	Yes (*n* = 105)	
	Count (% of Total)	Count (% of Total)	Count (% of Total)	
Glucose level				0.967 ^C^
≥11.1 mmol/L	19 (10.561)	8 (10.67)	11 (10.48)	
<11.1 mmol/L	161 (89.44)	67 (89.33)	94 (89.52)	
Serum sodium				0.449 ^C^
Normal	150 (83.33)	65 (86.67)	85 (80.95)	
Hypernatremia	12 (6.67)	3 (4.00)	9 (8.57)	
Hyponatremia	18 (10.00)	7 (9.33)	11 (10.48)	
Increased white blood cells				0.748 ^C^
No	60 (33.33)	24 (32.00)	36 (34.29)	
Yes	120 (66.67)	51 (68.00)	69 (65.71)	
Hypercoagulable disorder				0.751 ^C^
No	153 (85.00)	63 (84.00)	90 (85.71)	
Yes	27 (15.00)	12 (16.00)	15 (14.29)	
	**Mean** ± **SD (IQR)**	**Mean** ± **SD (IQR)**	**Mean** ± **SD (IQR)**	
Serum sodium (mmol/L)	139.60 ± 4.77 (119–150)	139.33 ± 4.07 (126–148)	139.8 ± 5.23 (119–150)	0.1926 ^M^
Glucose level (mmol/L)	80.31 ± 2.84 (5–24)	8.18 ± 2.28 (5–17)	8.40 ± 3.19 (5–24)	0.8788 ^M^
White blood cell (G/L)	12.56 ± 4.65 (3.1–28.67)	12.45 ± 4.34 (5.92–26.5)	12.64 ± 4.88 (3.1–28.67)	0.8891 ^M^

SD: standard deviation; IQR: Interquartile range; ^C^: *χ*^2^ test; ^M^: Mann–Whitney U test.

**Table 3:** Computed tomography (CT) imaging features in the in-hospital survival and in-hospital mortality patients with ICH.

Variables	All Patients (*n* = 180)	In-Hospital Mortality		*p*-Value
		No (*n* = 75)	Yes (*n* = 105)	
	Count (% of Total)	Count (% of Total)	Count (% of Total)	
Intracerebral hematoma location				0.568 ^F^
Supratentorial	149 (82.78)	60 (80.00)	89 (84.76)	
Cerebellum	8 (4.44)	3 (4.00)	5 (4.76)	
Brain stem	23 (12.78)	12 (16.00)	11 (10.48)	
Volume of intracerebral hematoma				<0.001 ^C,^***
Small	100 (55.56)	56 (74.67)	44 (41.90)	
Large	80 (44.44)	19 (25.33)	61 (58.10)	
Compression of the ventricular system				0.020 ^C,^*
No	42 (23.33)	24 (32.00)	18 (17.14)	
Yes	138 (76.67)	51 (68.00)	87 (82.86)	
Midline shift				0.064 ^C^
No	72 (40.00)	36 (48.00)	36 (34.29)	
Yes	108 (60.00)	39 (52.00)	69 (65.71)	
Brain herniation				<0.001 ^C,^***
No	131 (72.78)	70 (93.33)	61 (58.10)	
Yes	49 (27.22)	5 (6.67)	44 (41.90)	
Hemorrhagic ventricular dilatation				0.497 ^C^
No	142 (78.89)	61 (81.33)	81 (77.14)	
Yes	38 (21.11)	14 (18.67)	24 (22.86)	
Pneumonia				0.015 ^C,^*
No	12 (6.67)	1 (1.33)	11 (10.48)	
Yes	168 (93.33)	74 (98.67)	94 (89.52)	
Recurrence of bleeding				0.018 ^C,^*
No	135 (75.00)	63 (84.00)	72 (68.57)	
Yes	45 (25.00)	12 (16.00)	33 (31.43)	

SD: standard deviation; IQR: Interquartile range; ^F^: Fisher’s exact test; ^C^: *χ*^2^ test; * and ***: significant at 0.05 and 0.001, respectively.

**Table 4:** Clinical data before intubation in the in-hospital survival and in-hospital mortality patients with ICH.

Variables	All Patients (*n* = 180)	In-Hospital Mortality		*p*-Value
		No (*n* = 75)	Yes (*n* = 105)	
	Count (% of total)	Count (% of Total)	Count (% of Total)	
Blood pressure ≥ 180/100 (mmHg)	29 (16.11)	5 (6.67)	24 (22.86)	0.004 ^C,^**
GCS score				<0.001 ^F,^***
≤8	98 (54.44)	19 (25.33)	79 (75.24)	
9–12	71 (39.44)	48 (64.00)	23 (21.90)	
13–15	11 (6.11)	8 (10.67)	3 (2.86)	
Vertical oculocephalic reflex				<0.001 ^C,^***
Positive	83 (46.11)	60 (80.00)	23 (21.90)	
Negative	97 (53.89)	15 (20.00)	82 (78.10)	
Horizontal oculocephalic reflex				<0.001 ^C,^***
Positive	102 (56.67)	57 (76.00)	45 (42.86)	
Negative	78 (43.33)	18 (24.00)	60 (57.14)	
Pupillary reactivity				<0.001 ^C,^***
One pupil	36 (20.00)	7 (9.33)	29 (27.62)	
Both pupils	30 (16.67)	1 (1.33)	29 (27.62)	
Normal	114 (63.33)	67 (89.33)	47 (44.76)	
Pupillary light reflex				<0.001 ^C,^***
Positive	110 (61.11)	66 (88.00)	44 (41.90)	
Negative	70 (38.89)	9 (12.00)	61 (58.10)	
Indication for intubation				<0.001 ^C,^***
Respiratory	52 (28.89)	39 (52.00)	13 (12.38)	
Neurology	128 (71.11)	36 (48.00)	92 (87.62)	
	**Mean** ± **SD (IQR)**	**Mean** ± **SD (IQR)**	**Mean** ± **SD (IQR)**	
GCS score	8.31 (2.88)	10.24 (2.14)	6.93 (2.54)	<0.001 ^M,^***
A time interval from clinical onset to intubation	3.68 ± 4.08 (0.04–21)	4.72 ± 4.94 (0.04–21)	2.93 ± 3.14 (0.04–14)	0.032 ^M,^*

SD: standard deviation; IQR: Interquartile range; ^F^: Fisher’s exact test; ^C^: *χ*^2^ test; ^M^: Mann–Whitney U test, *, **, ***: significant at 0.05, 0.01, and 0.001, respectively.

**Table 5:** Multivariate logistic regression analysis of mortality risk factors for patients with intracranial hemorrhage undergoing endotracheal intubation.

Variables	OR	SE	95% CI	*p*-Value
Age	1.029	0.020	0.990–1.071	0.144
Gender (vs. male)				
Female	2.014	1.318	0.558–7.264	0.284
BMI > 25 (vs. no)				
Yes	3.752	3.970	0.471–29.851	0.211
Hypertension (vs. no)				
Yes	0.518	0.331	0.148–1.816	0.304
History of alcohol abuse (vs. no)				
Yes	1.541	1.041	0.410–5.795	0.522
Diabetes (vs. no)				
Yes	0.763	0.785	0.101–5.739	0.793
History of cerebrovascular accident (vs. no)				
Yes	4.087	3.546	0.746–22.390	0.105
Time interval between the onset and arrival to our center	1.047	0.252	0.653–1.680	0.846
Hyperglycemia (vs. no)				
Yes	4.161	4.157	0.587–29.485	0.154
Serum sodium (vs. normal)				
Hypernatremia	6.566	8.259	0.558–77.259	0.135
Hyponatremia	10.364	9.995	1.565–68.615	0.015 *
Increased white blood cells (vs. no)				
Yes	0.356	0.225	0.103–1.231	0.103
Hypercoagulable disorder (vs. no)				
Yes	0.568	0.440	0.124–2.591	0.466
Hematoma location (vs. supratentorial)				
Cerebellum	0.653	1.072	0.026–16.279	0.796
Brain stem	0.123	0.132	0.015–1.008	0.051
Volume of intracerebral hematoma (vs. small)				
Large	1.225	0.719	0.387–3.874	0.73
Compression of the ventricular system (vs. no)				
Yes	0.407	0.396	0.060–2.740	0.356
Midline shift (vs. no)				
Yes	1.555	1.379	0.273–8.851	0.619
Brain herniation (vs. no)				
Yes	19.807	18.249	3.255–120.522	0.001 **
Hemorrhagic ventricular dilatation (vs. no)				
Yes	2.314	1.605	0.593–9.017	0.227
Recurrence of bleeding (vs. no)				
Yes	0.385	0.280	0.092–1.603	0.19
Blood pressure ≥ 180/100 (mmHg) (vs. no)				
Yes	2.377	2.103	0.419–13.467	0.328
CGS score	0.518	0.095	0.361–0.744	<0.001 ***
Vertical oculocephalic reflex (vs. positive)				
Negative	6.336	3.982	1.848–21.720	0.003 **
Horizontal oculocephalic reflex (vs. positive)				
Negative	1.124	0.795	0.280–4.499	0.869
Pupillary reactivity (vs. one pupil)				
Both pupils	4.796	7.633	0.211–108.571	0.325
Normal	2.216	2.435	0.257–19.102	0.469
Pupillary light reflex (vs. positive)				
Negative	0.982	1.034	0.124–7.735	0.986
Indication for intubation (vs. respiratory)				
Neurology	2.154	1.605	0.500–9.279	0.303
A time interval from clinical onset to intubation	0.909	0.087	0.753–1.098	0.325
Pseudo R2	0.5334			

OR: odd ratio; SE: standard error; 95% CI: confidence interval 95%, *, **, ***: significant at 0.05, 0.01, and 0.001, respectively.

**Table 6:** A reduced model including the mortality risk factors for patients with intracranial hemorrhage undergoing endotracheal intubation: multivariate logistic regression analysis.

Variables	OR	SE	95% CI	*p*-Value
Patient age	1.031	0.018	0.995–1.068	0.083
Brain herniation (vs. no)				
Yes	10.268	6.902	2.749–38.344	0.001 **
Serum sodium (vs. normal)				
Hypernatremia	4.588	4.468	0.680–30.944	0.118
Hyponatremia	3.496	2.692	0.772–15.820	0.104
Vertical oculocephalic reflex (vs. positive)				
Negative	6.288	2.993	2.473–15.985	<0.001 ***
CGS score before endotracheal intubation	0.614	0.075	0.482–0.782	<0.001 ***
Hematoma location (vs. supratentorial)				
Infratentorial	0.348	0.216	0.102–1.180	0.09
Indication for intubation (vs. respiratory)				
Neurology	2.414	1.366	0.795–7.323	0.12
Hyperglycemia (vs. no)				
Yes	2.764	1.997	0.670–11.393	0.159
Pseudo R2	0.4798			

OR: odd ratio; SE: standard error; 95% CI: confidence interval 95%, ** and ***: significant at 0.01 and 0.001, respectively.

## Data Availability

The datasets generated and/or analyzed during the current study are available from the corresponding author on reasonable request.
